# JODS-PD: Single-Layer Primal–Dual Dynamical Optimization for IRS-Assisted NOMA Beamforming

**DOI:** 10.3390/s26165211

**Published:** 2026-08-17

**Authors:** Yitong Shi, Pei Peng, Zhiyuan Wang

**Affiliations:** 1 School of Communication and Information Engineering, Nanjing University of Posts and Telecommunications, Nanjing 210023, China; 2 School of Electrical and Automation Engineering, Nanjing Normal University, Nanjing 210023, China

**Keywords:** intelligent reflecting surface, non-orthogonal multiple access, primal–dual dynamical system, KKT equilibrium set, pseudo-transient continuation, beamforming

## Abstract

Joint active and passive beamforming in an intelligent reflecting surface (IRS)-assisted non-orthogonal multiple-access (NOMA) downlink is a non-convex constrained problem whose Karush–Kuhn–Tucker (KKT) points are generally non-isolated. We formulate the normalized beamformers, continuous IRS angles, and inequality multipliers as one coupled projected primal–dual dynamical state. Here, single-layer means simultaneous primal–dual evolution; the numerical integrator may still contain multiple stages. Angle coordinates remove the unit-modulus equalities, while an adjacent-chain representation reduces the received-power ordering constraints without changing their feasible set for a fixed decoding order. We prove equilibrium–KKT equivalence and local transverse exponential attraction of the KKT equilibrium set under strict complementarity, locally constant active rank, nonzero streams, a quotient second-order condition, and a sufficiently large finite penalty parameter. A stiff-ODE warm-up followed by a nonmonotone semismooth pseudo-transient continuation (PTC) method computes a steady-state root.

## 1. Introduction

An intelligent reflecting surface (IRS), also known as a reconfigurable intelligent surface, comprises many low-power elements whose reflection responses can be programmed electronically [1,2,3]. By reshaping the propagation environment rather than generating a new radio frequency signal, an IRS can improve coverage and spectral efficiency with less active hardware than a relay [4,5,6]. Early studies considered received-power maximization in point-to-point links and transmit-power minimization in multiuser multiple-input single-output downlinks [1,7]. These works established the benefit of joint active and passive beamforming while also exposing the main computational difficulty: the transmitter variables and unit-modulus reflection coefficients enter the effective channels multiplicatively [8].

Power-domain non-orthogonal multiple access (NOMA) superposes several user streams on the same time–frequency resource, and receivers recover them through successive interference cancellation (SIC) [9,10,11]. Its performance depends not only on individual signal-to-interference-plus-noise ratios but also on the decoding order and cross-decoding conditions. An IRS adds another tightly coupled design variable: changing its phases alters both the absolute channel gains and the relative strengths that support SIC. A phase configuration that strengthens one user can therefore tighten another user’s decoding constraint. Even with a fixed decoding order, joint active and passive beamforming remains a constrained non-convex problem with fractional rates, unit-modulus variables, and coupled SIC inequalities.

Optimization-based IRS-NOMA research has addressed active–passive coupling through alternating optimization (AO), successive convex approximation, semidefinite relaxation, manifold optimization, and learning-assisted designs [12,13,14]. Representative studies have jointly optimized transmit beamformers, IRS coefficients, power allocation, or decoding order under different channel and hardware assumptions [8,15,16,17,18,19]. Recent work has also extended resource allocation to STAR-RIS architectures [20,21]. Beyond IRS-NOMA, wireless sensor network studies have used quantum-assisted clustering and hybrid multi-objective metaheuristics to coordinate energy, routing, and link quality objectives [22,23]. These methods establish effective design routes, but their variable update architectures, feasibility mechanisms, and endpoint guarantees differ from the simultaneous primal–dual construction studied here.

A complementary line of work has clarified how IRS configuration, channel acquisition, and NOMA ordering interact. Existing studies show that practical performance depends not only on passive beamforming gain but also on the quality of cascaded channel information and the selected decoding order [15,16,17,18,24,25,26]. This motivates an optimization formulation that states its channel state information (CSI) assumptions explicitly and separates the discrete order choice from the continuous active–passive design.

Artificial intelligence is also changing the information exchanged and the decisions made in networked communication systems. At the propagation environment level, ADH-CLSNet applies cross-latitude spatiotemporal learning to atmospheric duct height diagnosis [27]. QCTF combines quantized communication with transferable feature fusion for bandwidth-limited multi-agent perception [28], whereas SIMAC integrates multimodal sensing with semantic communication [29]. In cooperative wireless learning, hierarchical model aggregation has been combined with adaptive grouping and resource allocation to reduce communication bottlenecks [30]. These developments point toward controllers that may process learned features or distributed model updates in addition to conventional CSI. Recent governance research has further introduced internal value development as a complementary perspective for aligning increasingly autonomous AI systems [31]. Within this broader landscape, the present study focuses on a transparent, model-based constrained optimization layer and its KKT behavior. To clarify the methodological distinctions, Table 1 compares JODS-PD with representative IRS–NOMA AO/SCA/SDR methods, MIPS, and AO-IPOPT in terms of variable updates, constraint treatment, endpoint interpretation, and implementation burden.

The mathematical ingredients used below—projected primal–dual systems, augmented Lagrangians, quotient space reasoning, and semismooth Newton/PTC steps—are established tools [32,33,34,35,36,37,38,39]. The contribution is their problem-specific integration for fixed-order IRS-assisted NOMA: the physical structure is reduced before the dynamics are formed, the non-isolated KKT set is analyzed transversely, and the resulting root system is solved and audited with active-set-aware numerical procedures.

The principal contributions are summarized as follows:

1. A simultaneous normalized primal–dual formulation is developed in which active beamformers, continuous IRS angles, and inequality multipliers evolve in one state. Single layer refers to this optimization architecture, rather than to the number of stages used by the numerical integrator.
2. The IRS unit-modulus equalities are removed exactly through angle coordinates, and the full received-power ordering family is replaced by an equivalent adjacent chain for a fixed decoding order. For the benchmark N = 2, M = 30, K = 4, this reduces the multiplier constraints from 31 to 19 and the real state dimension from 77 to 65.
3. Equilibrium–KKT equivalence and local transverse exponential attraction of the KKT equilibrium set are established under explicit regularity, strict complementarity, nonzero stream, quotient second-order, and sufficiently large finite penalty conditions. Phase orbit and multiplier fiber directions are treated as neutral tangent directions rather than incorrectly requiring the full Jacobian to be Hurwitz.
4. A stiff-ODE warm-up and a nonmonotone semismooth PTC root solver are combined with exact active-branch derivatives, structured Jacobian assembly, inactive multiplier elimination, and decomposed endpoint checks for primal feasibility, stationarity, dual feasibility, and complementarity.
5. The empirical evaluation comprises 30 channels and includes a same-model two-block alternating optimization IPOPT (AO-IPOPT) baseline, all 24 decoding orders on representative channels, multiple starts, parameter and conditioning tests, finite-size scalability up to a 242-dimensional state, fairness metrics, and imperfect CSI stress tests.

The remainder of the paper is organized as follows. Section 2 presents the IRS-NOMA model, CSI assumptions, fixed-order policy, and joint design problem. Section 3 develops the reduced primal–dual dynamics, equilibrium set analysis, and hybrid ODE–PTC solver. Section 4 reports the numerical study. Section 5 discusses practical implications, limitations, and extensions, and Section 6 concludes the paper.

## 2. System Model and Problem Formulation

### 2.1. IRS-Assisted Downlink

**Figure 1:**
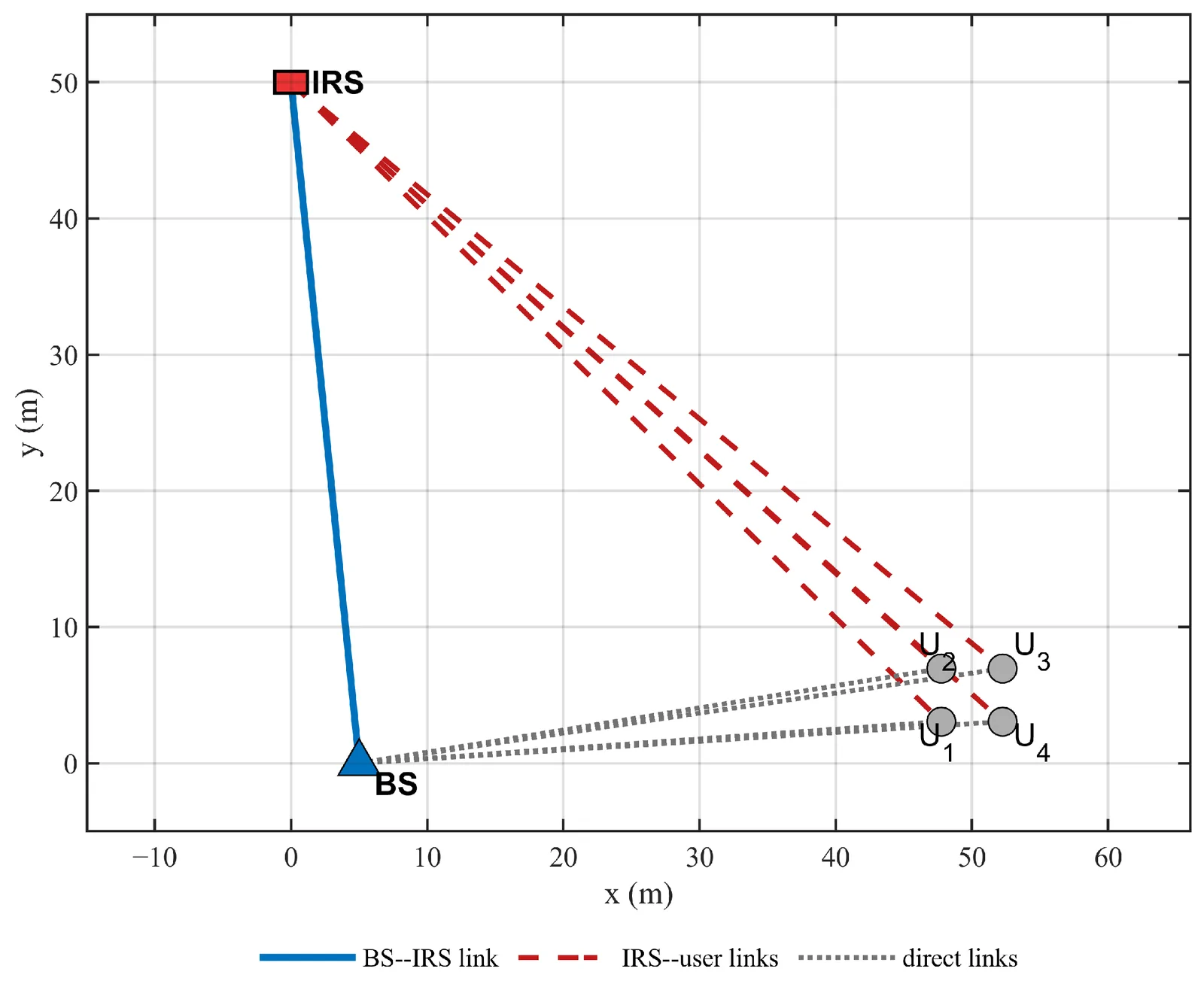
Top view of the simulated IRS-assisted NOMA downlink. The blue solid line denotes the BS–IRS link, the red dashed lines denote the IRS–user links, and the gray dotted lines denote the direct BS–user links. Together, the blue and red links form the two-hop BS–IRS–user paths.

We consider the downlink shown in Figure 1. A base station (BS) with N antennas serves K single-antenna users through direct links and a reflecting path provided by an IRS with M passive elements. The user set is K={1,…,K} and the IRS-element set is M={1,…,M}.

Let $h_{d,k}\in C^{N}$ denote the direct channel to user k, $G\in C^{M\times N}$ the BS–IRS channel, and $r_{k}\in C^{M}$ the IRS–user channel. The IRS response is

$$\Theta (\theta )=diag(\theta ),\;\;\theta_{m}=e^{j\phi_{m}},\;|\theta_{m}|=1.$$

The effective channel vector is

(1) $$h_{k}(\theta )=h_{d,k}+G^{H}\Theta^{H}(\theta )r_{k}.$$

The BS transmits the superposition-coded signal $x=\sum_{i=1}^{K}w_{i}s_{i}$, where $w_{i}\in C^{N}$ is the beamformer for stream i and $E|s_{i}{|}^{2}=1$. User k receives

(2) $$y_{k}=h_{k}^{H}(\theta )\sum_{i=1}^{K}w_{i}s_{i}+n_{k},\;\;n_{k}∼CN(0,\sigma^{2}).$$

### 2.2. Channel and Geometry Model

For a link of length d, the large-scale power gain is

(3) $$\beta (d)=\beta_{0}{\left(\frac{d}{d_{0}}\right)}^{-\alpha },$$

where $\beta_{0}$ is the reference gain at $d_{0}=1$ m, and α is link dependent. The obstructed BS–user channels are Rayleigh faded:

(4) $$h_{d,k}=\sqrt{\beta (d_{BU,k})}\;{\tilde{h}}_{d,k},\;\;{\tilde{h}}_{d,k}∼CN(0,I_{N}).$$

The BS–IRS and IRS–user channels are Rician faded:

(5) $$G=\sqrt{\beta (d_{BI})}\left(\sqrt{\frac{\kappa_{BI}}{\kappa_{BI}+1}}G_{LoS}+\sqrt{\frac{1}{\kappa_{BI}+1}}G_{NLoS}\right),$$

(6) $$r_{k}=\sqrt{\beta (d_{IU,k})}\left(\sqrt{\frac{\kappa_{IU}}{\kappa_{IU}+1}}r_{k,LoS}+\sqrt{\frac{1}{\kappa_{IU}+1}}r_{k,NLoS}\right).$$

The non-line-of-sight entries are independent CN(0,1) variables. For reproducibility, both the BS and IRS are modeled as horizontal uniform linear arrays with half-wavelength spacing. For an L-element array and azimuth ϑ,

$$a_{L}(\vartheta )=[1,e^{j\pi sin\vartheta },\ldots ,e^{j\pi (L-1)sin\vartheta }{]}^{T}.$$

The deterministic components are $G_{LoS}=a_{M}(\vartheta_{IB})a_{N}(\vartheta_{BI}{)}^{H}$ and $r_{k,LoS}=a_{M}(\vartheta_{Ik})$. Each azimuth is computed by atan2 from the endpoint-coordinate differences in Table 2. Users are drawn uniformly in area from the stated disk: the azimuth is uniform on [0,2π) and the radius is $r=3\sqrt{u}$ m with u∼U[0,1].

### 2.3. CSI Acquisition and Controller Architecture

The optimization layer assumes that one quasi-static estimate of the direct channels and the cascaded BS–IRS–user channels is available at a central controller during each coherence block. In an explicit CSI implementation, such estimates may be obtained through pilot-assisted cascaded channel estimation [16,24]. Implicit learning-based schemes can instead infer the RIS precoder directly from received pilots without an explicit channel estimation stage [25]. Interfacing such a scheme with the proposed optimizer would require the learned module to provide the effective channel quantities used in the optimization model. Under the explicit CSI architecture considered here, the controller computes the IRS phases, active beamformers, and decoding order, and then signals the resulting configuration to the BS, IRS controller, and users. The signaling and training overhead are not included in the reported solver times.

The perfect CSI results therefore quantify the optimization behavior conditional on the channel estimate. Section 4.9 separately perturbs the effective channels at prescribed normalized mean-square-error (NMSE) levels and evaluates feasibility on the corresponding true channels. This separation follows the established distinction between channel acquisition and resource optimization in IRS-assisted systems [24,25,26].

### 2.4. Fixed Decoding Order and SIC

The decoding order is selected once before the continuous optimization. Let the unit-modulus reference phase vector define effective reference-channel gains; users are ranked in nondecreasing order of these gains, with a deterministic user-index tie break. This produces a feasible and reproducible fixed-order policy while avoiding the K! combinatorial factor inside the continuous primal–dual state.

(7) $$∥h_{\pi_{1}}(\theta^{ref}){∥}^{2}\leq \cdots \leq ∥h_{\pi_{K}}(\theta^{ref}){∥}^{2},\;\;\Omega (\pi_{r})=r,$$

The same reference phase vector and resulting permutation are used to construct the initialization for all compared solvers. This policy is not claimed to be globally order-optimal. To quantify its effect, Section 4.7 enumerates all 24 orders for three representative channels and distinguishes the selected reference order from the best successful order found by enumeration.

When user j decodes stream k, with Ω(j)≥Ω(k), its signal-to-interference-plus-noise ratio is

(8) $$\Gamma_{k\to j}=\frac{|h_{j}^{H}w_{k}{|}^{2}}{\sum_{\Omega (i)>\Omega (k)}|h_{j}^{H}w_{i}{|}^{2}+\sigma^{2}},\;\;R_{k\to j}={log}_{2}(1+\Gamma_{k\to j}).$$

(9) $$R_{k\to j}\geq R_{k\to k},\;\;\Omega (j)>\Omega (k)$$

The achievable rate assigned to user k is $R_{k}=R_{k\to k}$. SIC requires each later-decoding user to support stream k at that rate.

Following the received-power ordering used in IRS-NOMA designs [13], define $q_{li}=|h_{l}^{H}w_{i}{|}^{2}$. The power of an earlier-decoded stream must be no smaller than that of a later-decoded stream at every receiver:

(10) $$q_{li}\leq q_{lj},\;\;l\in K,\;\Omega (i)>\Omega (j).$$

Constraint (10) is a received-power ordering condition. It is not a minimum-rate or rate-fairness constraint; consequently, sum-rate maximization can still produce unequal user rates.

### 2.5. Joint Design Problem

The beamformers and IRS coefficients are designed to maximize the downlink sum rate:

(11) $$\begin{array}{llll} (P1):\;\underset{\{w_{k}\},\;\theta }{max} & \sum_{k=1}^{K}R_{k} & & \\ \;\;\;\;s.t.\; & |\theta_{m}{|}^{2}=1, & m\in M, & \left(C1\right)\\ & \sum_{k=1}^{K}∥w_{k}{∥}^{2}\leq P_{T}, & & \left(C2\right)\\ & R_{k\to k}-R_{k\to j}\leq 0, & \Omega (j)>\Omega (k), & \left(C3\right)\\ & q_{li}-q_{lj}\leq 0, & l\in K,\;\Omega (i)>\Omega (j). & \left(C4\right) \end{array}$$

Problem (P1) is non-convex even with a fixed decoding order. The effective channels couple θ and $\left\{w_{k}\right\}$, (C1) is non-convex in the complex coefficients, and both the objective and (C3) are nonlinear fractional functions of the design. Section 3 removes (C1) by parameterization and treats the remaining inequalities with one primal–dual dynamical system.

## 3. Single-Layer Primal–Dual Dynamical Formulation

This section converts (P1) into an inequality-constrained real problem and constructs one coupled projected primal–dual system. In this paper, single-layer denotes simultaneous evolution of the primal and multiplier variables without an outer multiplier-update loop. It does not mean that the numerical realization has only one stage: the same residual map is first followed by a short stiff-ODE warm-up and is then solved by a semismooth PTC root iteration.

### 3.1. Angle Parameterization and Constraint Reduction

Write each beamformer as $w_{k}=\sqrt{P_{T}}\;{\bar{w}}_{k}$ and each IRS coefficient as $\theta_{m}=e^{j\phi_{m}}$. The real primal vector is

(12) $$y=[Re({\bar{w}}_{1}{)}^{T},Im({\bar{w}}_{1}{)}^{T},\ldots ,Re({\bar{w}}_{K}{)}^{T},Im({\bar{w}}_{K}{)}^{T},\phi^{T}{]}^{T}\in R^{n_{y}},\;\;n_{y}=2NK+M.$$

The unit-modulus constraints now hold identically, and the normalized power inequality is

(13) $$h^{pow}(y)=\sum_{k=1}^{K}∥{\bar{w}}_{k}{∥}^{2}-1\leq 0.$$

For every pair Ω(j)>Ω(k), define the SIC inequality

(14) $$h_{kj}^{sic}(y)=R_{k\to k}(y)-R_{k\to j}(y)\leq 0.$$

The received-power ordering constraints can be represented more compactly. For each observing user l, it is sufficient to impose only adjacent ranks:

(15) $$h_{lr}^{ord}(y)=\frac{q_{l,\pi_{r+1}}(y)-q_{l,\pi_{r}}(y)}{\sigma^{2}}\leq 0,\;\;r=1,\ldots ,K-1.$$

Division by the positive constant $\sigma^{2}$ does not alter feasibility; it brings the received-power differences, which are otherwise of order $10^{-12}$ in the tested geometry, to a useful numerical scale.

**Proposition** **1** (equivalence of adjacent ordering)**.** *The adjacent constraints (15) are equivalent to the full pairwise family (C4) for a fixed decoding order.*

**Proof.**  For a fixed observer l, (15) gives

$$q_{l,\pi_{1}}\geq q_{l,\pi_{2}}\geq \cdots \geq q_{l,\pi_{K}}.$$

Transitivity therefore gives $q_{l,\pi_{s}}\leq q_{l,\pi_{r}}$ for every s>r, which is (C4). Conversely, the full pairwise family contains every adjacent pair. □

Stack (13)–(15) into h(y)≤0 and set

$$f(y)=-\sum_{k=1}^{K}R_{k}(y).$$

The angle-parameterized problem is

(16) $$(P2):\;\;\underset{y\in R^{n_{y}}}{min}f(y)\;s.t.\;h(y)\leq 0.$$

There is one power inequality, K(K−1)/2 SIC inequalities, one for each user-rank pair satisfying Ω(j)>Ω(k), and K(K−1) adjacent ordering inequalities, corresponding to K observing users and K−1 adjacent rank pairs per observer. Therefore,

(17) $$p=1+\frac{K(K-1)}{2}+K(K-1).$$

For N = 2, M = 30, and K = 4, the adjacent-chain formulation uses 19 inequality multipliers, compared with 31 for the unreduced pairwise ordering family. The resulting real state dimension decreases from 77 to 65. Proposition 1 proves that this reduction changes neither the feasible set nor the fixed-order objective; it only removes transitive ordering inequalities and their multipliers.

### 3.2. JODS-PD Dynamics

Let $\mu \in R_{\geq 0}^{p}$ be the inequality multipliers and ρ>0 a penalty parameter. Define the projected multiplier as

(18) $$s(y,\mu )=[\mu +\rho h(y){]}_{+},$$

where the maximum with zero is applied componentwise. The inequality augmented Lagrangian in Hestenes–Powell–Rockafellar form is [32,33]

(19) $$A_{\rho }(y,\mu )=f(y)+\frac{1}{2\rho }\left(∥s(y,\mu ){∥}^{2}-∥\mu {∥}^{2}\right).$$

Let $J_{h}(y)=\partial h/\partial y\in R^{p\times n_{y}}$. The gradient of (19) with respect to the primal variables is

(20) $$\nabla_{y}A_{\rho }=\nabla f(y)+J_{h}(y{)}^{T}s(y,\mu ).$$

JODS-PD evolves the primal and dual variables simultaneously:

(21) $$\begin{array}{l} \dot{y}=-\nabla f(y)-J_{h}(y{)}^{T}s(y,\mu ),\\ \dot{\mu }=\frac{\gamma }{\rho }\left(s(y,\mu )-\mu \right), \end{array}\;\;\gamma >0.$$

If μ(0)≥0, the nonnegative orthant is invariant: at a boundary component $\mu_{i}=0$, ${\dot{\mu }}_{i}=(\gamma /\rho )s_{i}\geq 0$. The complete state is $z=[y^{T},\mu^{T}{]}^{T}\in R^{d}$, with $d=n_{y}+p$. For the benchmark dimensions N=2, M=30, and K=4, d=65. We write (21) compactly as $\dot{z}=F(z)$.

**Proposition** **2** (equilibrium–KKT equivalence)**.** *A state* $\left(y^{*},\mu^{*}\right)$ *with* $\mu^{*}\geq 0$ *is an equilibrium of (21) if and only if it satisfies the KKT conditions of (P2).*

**Proof.**  At an equilibrium, the dual equation gives

(22) $$\mu^{*}=[\mu^{*}+\rho h(y^{*}){]}_{+}.$$

Consider component i. If $\mu_{i}^{*}>0$, (22) implies $\mu_{i}^{*}=\mu_{i}^{*}+\rho h_{i}(y^{*})$, hence $h_{i}(y^{*})=0$. If $\mu_{i}^{*}=0$, (22) implies $h_{i}(y^{*})\leq 0$. Thus, primal feasibility, dual feasibility, and complementarity hold. The primal equation, together with $s=\mu^{*}$, gives

$$\nabla f(y^{*})+J_{h}(y^{*}{)}^{T}\mu^{*}=0,$$

which is stationarity. Conversely, KKT feasibility and complementarity imply (22), and KKT stationarity makes the primal equation zero. □

Proposition 2 is an equilibrium statement, not a global-convergence theorem. Because (P2) is non-convex, several KKT equilibria may exist and the one reached can depend on the channel realization and initialization.

### 3.3. Stability of the KKT Equilibrium Set

The projected map in (18) is nonsmooth, where $\mu_{i}+\rho h_{i}=0$. Strict complementarity places a KKT state away from this switching surface: active constraints have $\mu_{i}^{*}>0$, whereas inactive constraints have $h_{i}(y^{*})<0$ and $\mu_{i}^{*}=0$. The projected flow is therefore smooth on a neighborhood with a fixed active set.

A second issue is specific to the complex beamformers. The objective and every constraint depend on stream i through squared magnitudes such as $|h_{j}^{H}w_{i}{|}^{2}$. They are unchanged by the independent rotations ${\bar{w}}_{i}\mapsto e^{j\alpha_{i}}{\bar{w}}_{i}$. If all ${\bar{w}}_{i}^{*}$ are nonzero, the torus $T^{K}$ generates a K-dimensional orbit of physically equivalent KKT points. In the real coordinates of (12), its tangent space is spanned by vectors whose stream-i block is

$$t_{i}=[-Im({\bar{w}}_{i}^{*}{)}^{T},Re({\bar{w}}_{i}^{*}{)}^{T}{]}^{T},$$

with zeros in the other beamformer and IRS-angle blocks. Denote this phase-tangent space by $T_{\phi }$. Pointwise asymptotic stability is impossible because every neighborhood of one equilibrium contains other equilibria on the same orbit [34].

Let $A=\{i:h_{i}(y^{*})=0\}$, let $J_{A}$ contain the active constraint gradients, and define the Lagrangian Hessian as

(23) $$H_{L}=\nabla^{2}f(y^{*})+\sum_{i\in A}\mu_{i}^{*}\nabla^{2}h_{i}(y^{*}).$$

The relevant feasible directions are those orthogonal to the phase orbit. Define the quotient critical subspace as

$$C_{q}=ker(J_{A})\cap T_{\phi }^{⟂}.$$

If $J_{A}$ has rank r<|A|, active multipliers are also nonunique: perturbations in ker$\left(J_{A}^{T}\right)$ leave stationarity unchanged. Locally, the equivalent KKT states therefore comprise the phase orbit together with this dual fiber. We denote that set by E.

**Theorem** **1** (local transverse exponential attraction of the KKT equilibrium set)**.** Let z* = (y*, μ*) be a KKT state of (P2). Assume that (i) every stream beamformer is nonzero; (ii) strict complementarity holds on the selected projected branch; (iii) the active-constraint Jacobian has locally constant rank; (iv) the phase orbit tangent space, multiplier fiber tangent space, and their complementary normal spaces have locally constant dimensions and admit a smooth direct-sum decomposition; and (v) the Lagrangian Hessian is positive definite on the feasible quotient-critical subspace obtained after removing the phase symmetry directions.

$$d^{T}H_{L}d>0,\;\;0\neq d\in C_{q},$$

*Then there exists a finite threshold ρ_0_ such that, for every finite ρ > ρ_0_ and every γ > 0, the local KKT equilibrium set ℰ is transversely exponentially attractive. Specifically, for all initial conditions sufficiently close to ℰ, the continuous-time trajectory remains local and satisfies dist(z(t), ℰ) ≤ C exp(−ct) dist(z(0), ℰ) for some C ≥ 1 and c > 0. The statement concerns local continuous-time behavior of the projected dynamics; it is not a global convergence theorem for the hybrid ODE* *–PTC numerical solver.*

**Proof.**  Strict complementarity fixes the local branch of the projected map, so the right-hand side is continuously differentiable in a neighborhood of z*. The locally constant active rank and the constant dimension assumptions imply, by the constant rank theorem, that the nearby KKT states form a smooth local manifold. Its tangent space contains the per-stream phase orbit and, when the active gradients are rank-deficient, the multiplier fiber ker(Aᵀ).

$$Q_{\rho }=H_{L}+\rho J_{A}^{T}J_{A}$$

is positive definite on $T_{\phi }^{⟂}$ and has kernel $T_{\phi }$ [32,33]. Under strict complementarity, the active linearization is

(24) $$\left[\begin{array}{c} \delta \dot{y}\\ \delta {\dot{\mu }}_{A} \end{array}\right]=\left[\begin{array}{cc} -Q_{\rho } & -J_{A}^{T}\\ \gamma J_{A} & 0 \end{array}\right]\left[\begin{array}{c} \delta y\\ \delta \mu_{A} \end{array}\right].$$

Decompose primal perturbations into phase-tangent and phase-normal components and active-multiplier perturbations into the multiplier fiber and its orthogonal complement. The quotient second-order condition makes the reduced Lagrangian Hessian positive definite on feasible phase-normal directions. For a sufficiently large but finite penalty parameter, the augmented normal block Qρ is therefore positive definite on the full complementary subspace.

$$V=\frac{1}{2}∥\delta y_{⟂}{∥}^{2}+\frac{1}{2\gamma }∥\delta \mu_{A,⟂}{∥}^{2}.$$

On that complementary subspace, the linearized primal–dual block has a Hurwitz spectrum for every γ > 0: γ changes the relative primal–dual time scales and conditioning but not the sign of the transverse real parts. The only local zero modes are tangent to the phase orbit and multiplier fiber. Standard normally hyperbolic invariant manifold arguments then yield exponential decay of the distance to ℰ, which proves the stated set attraction property [37,39]. □

Accordingly, the full Jacobian is not expected to be Hurwitz. Its neutral eigenvalues encode physical phase invariance and active-multiplier nonuniqueness, whereas the nonzero transverse eigenvalues determine local attraction to the equilibrium set. Section 4.5 evaluates these quantities numerically and checks that the observed zero-mode count agrees with the predicted tangent dimension.

### 3.4. Hybrid ODE–PTC Solver

#### 3.4.1. Shared Reference Phase and Initialization

For a channel seed, a reference vector is sampled as $\theta_{m}^{ref}=e^{j\varphi_{m}}$ with $\varphi_{m}∼U[0,2\pi )$. It defines the fixed order in (7) and is also used as the initial IRS vector. Using one reference vector prevents a mismatch between the fixed order and the common solver start [40,41].

The initial beamformers share one direction. Let $\pi_{1}$ be the first-decoded user and set

(25) $$v=\frac{h_{\pi_{1}}(\theta^{ref})}{∥h_{\pi_{1}}(\theta^{ref})∥},\;\;p_{k}=P_{T}\frac{1/\Omega (k)}{\sum_{i}1/\Omega (i)},\;\;w_{k,0}=\sqrt{p_{k}}v.$$

This start obeys the power budget and the received-power order because all streams have the same spatial direction and $p_{\pi_{1}}\geq \cdots \geq p_{\pi_{K}}$. It does not need to satisfy every SIC inequality; feasibility is established by the primal–dual trajectory.

#### 3.4.2. Stiff-ODE Warm-Up

Starting PTC directly from the shared physical initialization can produce a large first root correction because the multiplier active set changes rapidly near the initial point. The short ode15s phase follows the same continuous projected field, preserves μ ≥ 0, and moves the state toward a branch on which the generalized Jacobian is more informative. It is therefore a numerical warm-up rather than a distinct optimization layer.

#### 3.4.3. Active-Set Semismooth Pseudo-Transient Continuation

After warm-up, PTC seeks a root F(z) = 0. The positive-part map is strongly semismooth, and its derivative is exact on each fixed active branch. We assemble the corresponding generalized Jacobian by automatic differentiation, refresh it periodically or when the active branch changes, and solve the shifted PTC system for the root correction.

(26) $$\left(-\frac{I}{\sigma_{k}}+V_{k}\right)d_{k}=F(z_{k}),\;\;z_{k+1}=z_{k}-d_{k}.$$

For a small $\sigma_{k}$, the diagonal term regularizes the step and resembles implicit time integration. As $\sigma_{k}$ grows, (26) approaches a Newton step for the equilibrium equation. The initial value follows the AQGS scaling used in [42,43],

(27) $$\sigma_{0}=c\frac{\sqrt{\varepsilon_{b}}}{max(∥F(z_{0}){∥}_{2},10^{-12})},\;\;c=0.6,\;\varepsilon_{b}=10^{-2},$$

and subsequent values use the switched evolution–relaxation update

(28) $$\sigma_{k+1}=\sigma_{k}\frac{∥F(z_{k}){∥}_{2}}{max(∥F(z_{k+1}){∥}_{2},10^{-300})}.$$

The residual update is intentionally nonmonotone. A trial step may be accepted even when the residual temporarily rises, because branch transitions can require leaving the current local model before the PTC step enters a better-conditioned neighborhood. Refresh events are classified as scheduled, active-set-triggered, or near-root in Section 4.2.

Unlike the continuous flow, a Newton-like PTC correction is not an invariant dynamical trajectory and can temporarily leave the nonnegative multiplier orthant. In the four representative runs, the most negative intermediate multiplier ranged from approximately 0 to −5.18 × 10^−3^, while all accepted endpoints satisfied dual feasibility and complementarity. Directly projecting every correction preserves nonnegativity but changes the semismooth Newton direction; it solved two of three tested channels, whereas the difficult channel did not meet the 2500-iteration criterion. We therefore retain the unprojected root correction and require independent endpoint checks of primal feasibility, stationarity, dual feasibility, and complementarity.

#### 3.4.4. Algorithm

Based on the preceding development, the complete computational procedure of JODS-PD is summarized in Algorithm 1.
**Algorithm 1.** JODS-PD with stiff-ODE warm-up and semismooth PTCInput: one channel estimate; N, M, K; reference-phase seed; ρ, γ; ODE/PTC settings; residual tolerances. Output: beamformers, IRS phases, inequality multipliers, fixed decoding order, and decomposed endpoint residuals.Draw the reference phases, determine the fixed order, and construct the shared physical initialization.Form the reduced objective, constraints, projected multipliers, and residual map F(z).Generate exact smooth-piece derivatives and the active-branch generalized Jacobian.Integrate the projected flow briefly with ode15s; extend the warm-up only when the pilot PTC residual exceeds the prescribed threshold.Initialize the PTC step length and active branch.Solve the shifted PTC linear system; update the step length by switched evolution–relaxation.Refresh the generalized Jacobian on schedule, after an active-set change, or near the root.Stop when the unified flow/KKT and feasibility thresholds are met, or when the iteration limit is reached.Recover the physical variables and report primal feasibility, stationarity, dual feasibility, complementarity, sum rate, user rates, and solver status.

### 3.5. Computational Cost

The dynamical state has

(29) $$d=2NK+M+1+\frac{K(K-1)}{2}+K(K-1)$$

real components. A dense factorization of the shifted system costs O(d^3^) arithmetic operations and O(d^2^) memory per refresh, while residual and derivative evaluation depends on the channel dimensions and active set. These expressions describe the present finite-dimensional implementation rather than an asymptotic complexity advantage over all competing methods.

The implementation exploits two structures that become important as d grows: the Jacobian is assembled in exact primal–dual blocks, and multipliers that are strictly inactive on the current branch are eliminated from the shifted linear solve and then recovered explicitly. Section 4.8 compares this structured realization with the dense assembly on pre-specified finite-size instances.

## 4. Numerical Results

### 4.1. Reproducible Comparison Protocol

The numerical study used the geometry and channel model in Table 2. Thirty independent channel realizations were used for distributional statistics. Seeds 42–51 were used for the matched JODS-PD/AO-IPOPT/MIPS comparison, and seeds 42, 54, and 58 were used for the order, multi-start, sensitivity, and conditioning studies. Saved channel realizations were reused across methods so that all paired comparisons had identical physical inputs.

For each paired run, all methods received the same channel, initial physical vector, and fixed decoding order. JODS-PD and the two-block alternating optimization IPOPT (AO-IPOPT) baseline used the same angle-parameterized model and exact derivatives. The AO baseline alternated an active beamforming block and an IRS-angle block, solved each block with IPOPT, and stopped when the relative outer objective change was below 10^−7^ and the maximum constraint violation was below 10^−6^, subject to 50 outer iterations. MATPOWER Interior Point Solver (MIPS) used the full nonlinear formulation and the termination criteria documented in its algorithm description and user manual [44,45].

All smooth-piece derivatives were generated by CasADi 3.7.2 [46]. An independent Python/CasADi evaluation of a saved MATLAB R2023b state reproduced the objective and constraints with a maximum absolute difference of 1.31 × 10^−10^. The tests were executed serially on the same workstation; saved per-run records include returned objectives, user rates, iteration counts, timing components, constraint residuals, and KKT diagnostics.

Unless a test stated otherwise, a run was successful when the maximum scaled constraint violation was at most 10^−6^, the flow/KKT infinity norm was at most 10^−6^, the dual-feasibility residual was at most 10^−8^, and complementarity was numerically satisfied. Because MIPS uses a different internal stopping rule, its endpoints were evaluated separately against the common 10^−6^ primal feasibility criterion. Distributional summaries report the mean, standard deviation, median, interquartile range (IQR), and range where informative. The principal numerical settings and termination criteria used for JODS-PD and MIPS are summarized in Table 3.

### 4.2. Convergence and Feasibility of JODS-PD

**Figure 2:**
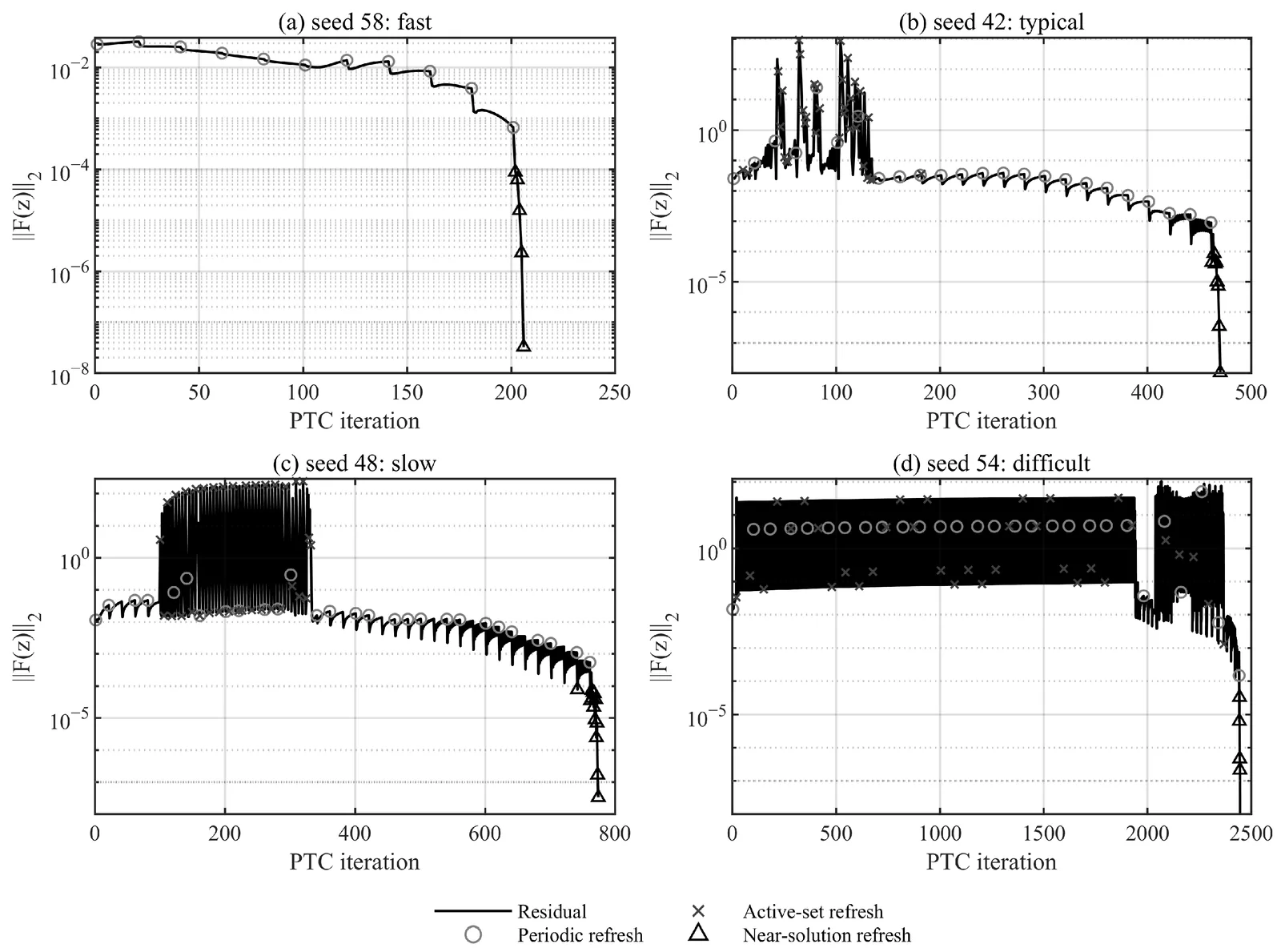
JODS-PD convergence for four representative channels. Panels (**a**–**d**) show the flow/KKT residual histories for seeds 58, 42, 48, and 54, respectively. Circles mark scheduled or periodic generalized Jacobian refreshes, crosses mark active-set-triggered refreshes, and triangles mark near-root refreshes. The nonmonotone middle phases correspond to accepted PTC branch transitions.

Figure 2 compares four representative convergence behaviors: fast (seed 58), typical (seed 42), slow (seed 48), and difficult (seed 54). All reached the unified endpoint criterion, but their iteration counts and generalized-Jacobian refresh patterns differed substantially. The difficult case spent most iterations adapting to repeated active-branch changes before entering a short near-root phase.

The residual histories are not required to decrease at every iteration. The switched evolution update can enlarge the PTC step while the active branch is still changing; after the branch stabilizes, the shifted system approaches a semismooth Newton step and the residual drops rapidly. This behavior explains why a single representative trajectory understates the observed variation in numerical difficulty.

The corresponding iteration counts were 206, 470, 774, and 2446. Their final flow-residual infinity norms were 1.54 × 10^−8^, 5.30 × 10^−9^, 2.17 × 10^−8^, and 2.79 × 10^−11^, respectively. Table 4 separates refresh events by cause and shows that active-set-triggered refreshes dominated only in the difficult case.

### 4.3. Matched Three-Method Comparison

Table 5 summarizes the ten matched benchmark channels. JODS-PD satisfied the full endpoint criterion in all ten cases. AO-IPOPT reached its outer convergence and primal feasibility criteria in all ten cases, whereas MIPS met the common 10^−6^ primal feasibility criterion in eight. The sum rate comparison is therefore made on the eight channels common to all three methods.

On the eight common successful channels, the median returned sum rates were 5.7285, 5.6923, and 5.2145 bit/s/Hz for JODS-PD, AO-IPOPT, and MIPS, respectively. JODS-PD and AO-IPOPT optimize the same angle-parameterized model with exact derivatives, so their close medians provide a direct same-model cross-check. The remaining difference is consistent with convergence to different local stationary points in a non-convex problem.

**Figure 3:**
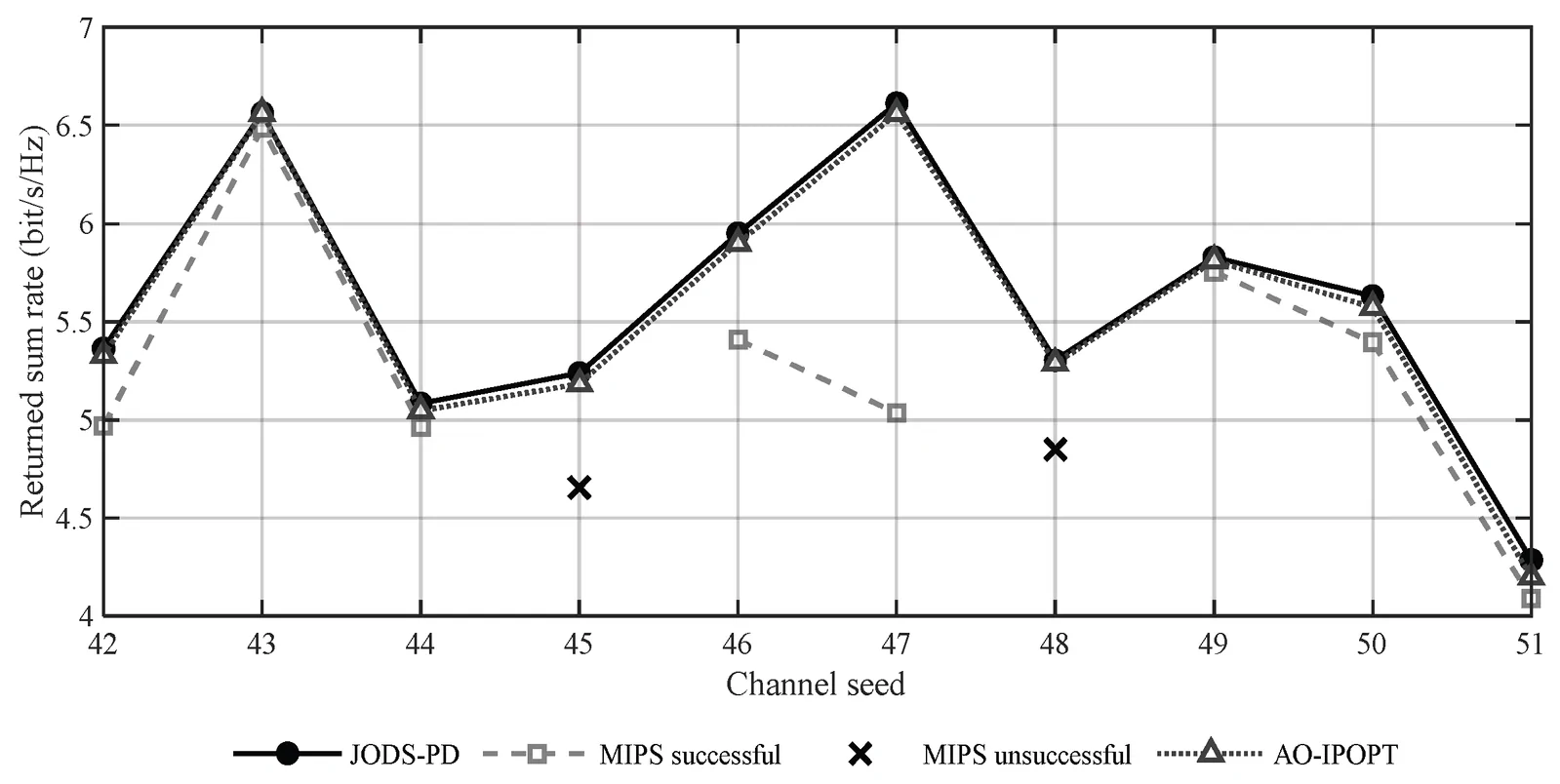
Per-seed returned sum rate for JODS-PD, AO-IPOPT, and MIPS on the matched benchmark channels. Open or missing MIPS markers identify endpoints that do not meet the common 10^−6^ primal feasibility criterion.

Endpoint quality was also assessed at multiple KKT thresholds. Among the ten JODS-PD endpoints, 10, 10, and 10 satisfy thresholds of 10^−5^, 10^−6^, and 10^−7^, respectively. The corresponding MIPS counts were 6, 5, and 1 after reconstructing the common stationarity and complementarity diagnostics. These values describe the returned endpoints under one common audit and complement, rather than replace, each solver’s native termination information. Figure 3 further compares the per-seed returned sum rates of JODS-PD, AO-IPOPT, and MIPS and identifies the MIPS endpoints that do not satisfy the common primal-feasibility criterion.

MIPS did not satisfy the stated primal feasibility criterion on seeds 45 and 48. AO-IPOPT reached outer convergence and primal feasibility on all ten matched seeds and on 28 of the 30 channels. Its reconstructed full-KKT stationarity residual was approximately 7 × 10^−3^ on the matched cases, so it is reported as a feasible block-stable baseline rather than as a full-root solver.

**Figure 4:**
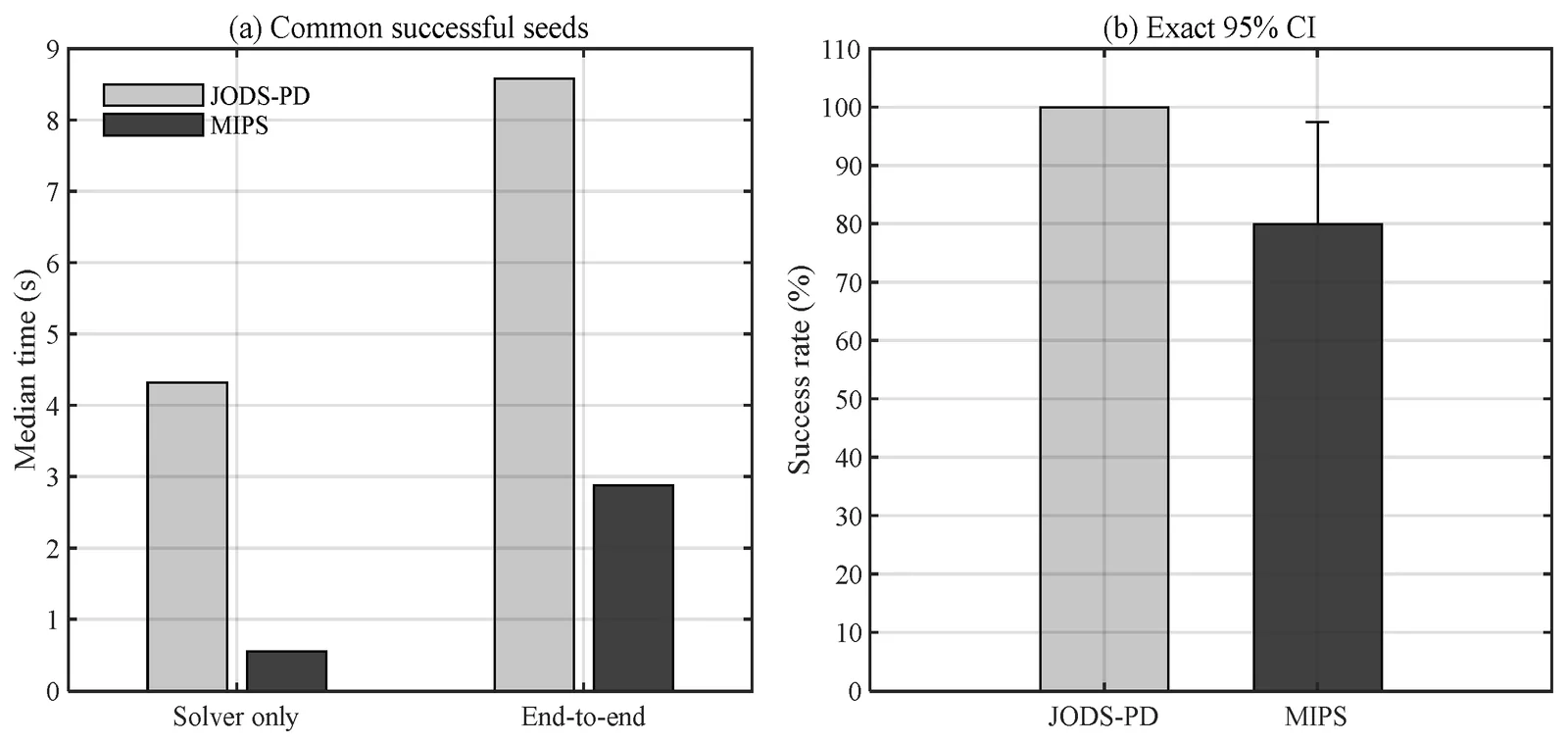
JODS-PD and MIPS reliability and timing. (**a**) Median solver-only and end-to-end wall times on eight common successes; (**b**) success rates with exact 95% binomial confidence intervals.

Figure 4 complements the per-seed objective values with success frequencies and solver-time distributions. Because the methods implement different numerical architectures, the figure reports measured times together with endpoint status; the primary comparison is the combination of returned objective, feasibility, and KKT quality.

JODS-PD incurs active-branch derivative assembly and shifted linear solves, AO-IPOPT repeatedly solves two nonlinear blocks, and MIPS applies a generic interior-point formulation. These architectural differences explain the timing pattern. The experiments support the ability of JODS-PD to return low-residual KKT endpoints consistently on the tested channels; they do not imply a universal timing advantage for every implementation or system dimension.

### 4.4. User-Rate Allocation and Objective Scope

**Figure 5:**
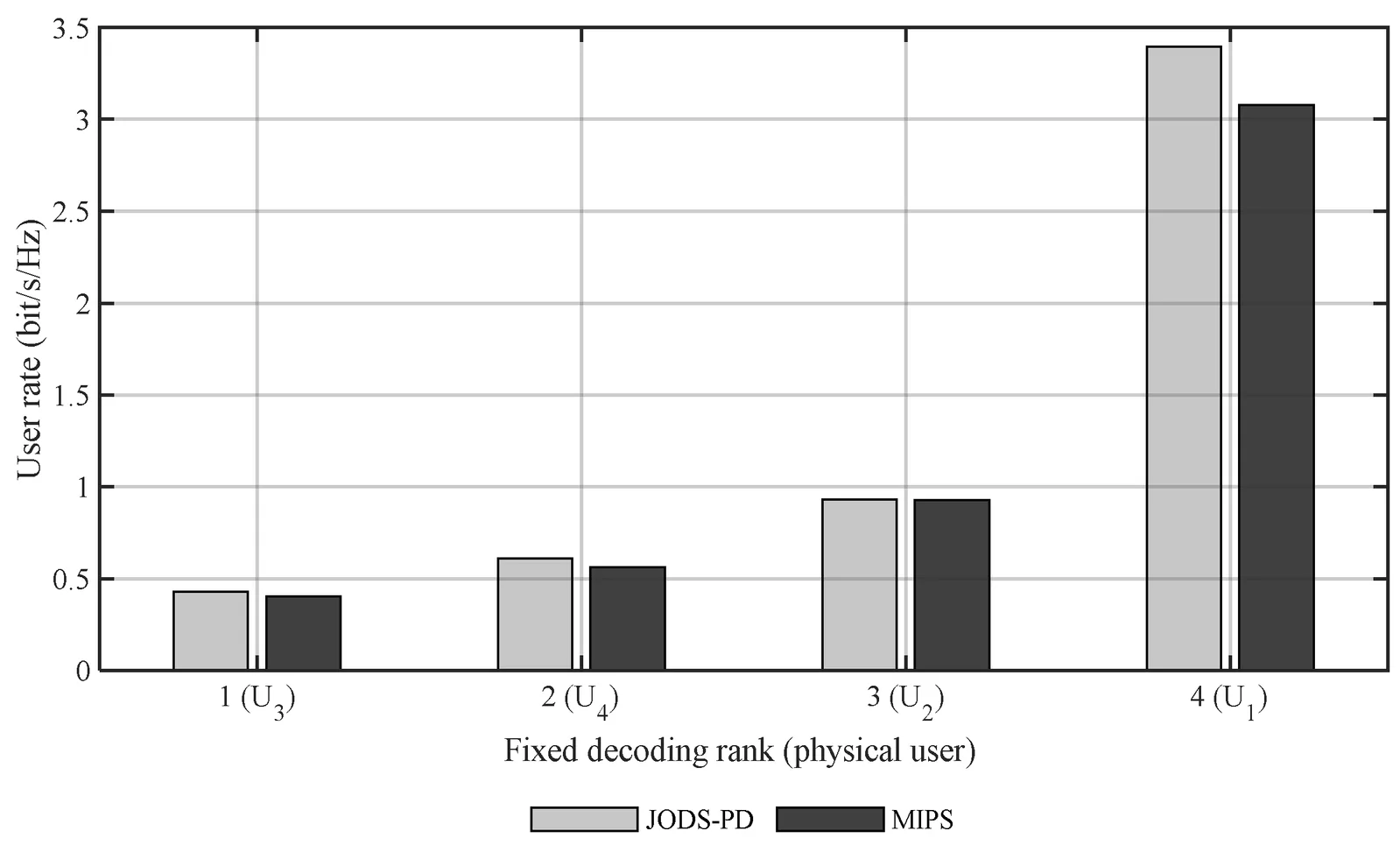
User-rate allocation for seed 42, sorted by the fixed decoding rank. Grayscale-safe bars distinguish JODS-PD and MIPS; the distribution reflects the sum-rate objective without explicit minimum-rate constraints.

Figure 5 reports the user-rate allocation for seed 42, sorted by the fixed decoding rank. Across the 30-channel JODS-PD study, the minimum-user rate has mean ± standard deviation 0.4188 ± 0.0193 bit/s/Hz and median 0.4148 (IQR 0.4085–0.4251); the Jain index has mean ± standard deviation 0.5399 ± 0.0498 and median 0.5248 (IQR 0.5072–0.5582). These metrics describe the allocation produced by sum-rate maximization and are not additional optimization constraints.

The fairness metrics confirm the intended scope of (P1): feasibility, received-power ordering, and sum-rate maximization are enforced, whereas minimum-user-rate and fairness requirements are not. Such requirements can be added as smooth inequalities R_k_ ≥ R_k,min_ or through a weighted utility; the projected primal–dual construction then gains the corresponding multipliers without changing its single-layer architecture. The distributional statistics of the sum rate, minimum-user rate, Jain index, PTC iterations, and solver time over the 30 channel realizations are summarized in Table 6.

**Figure 6:**
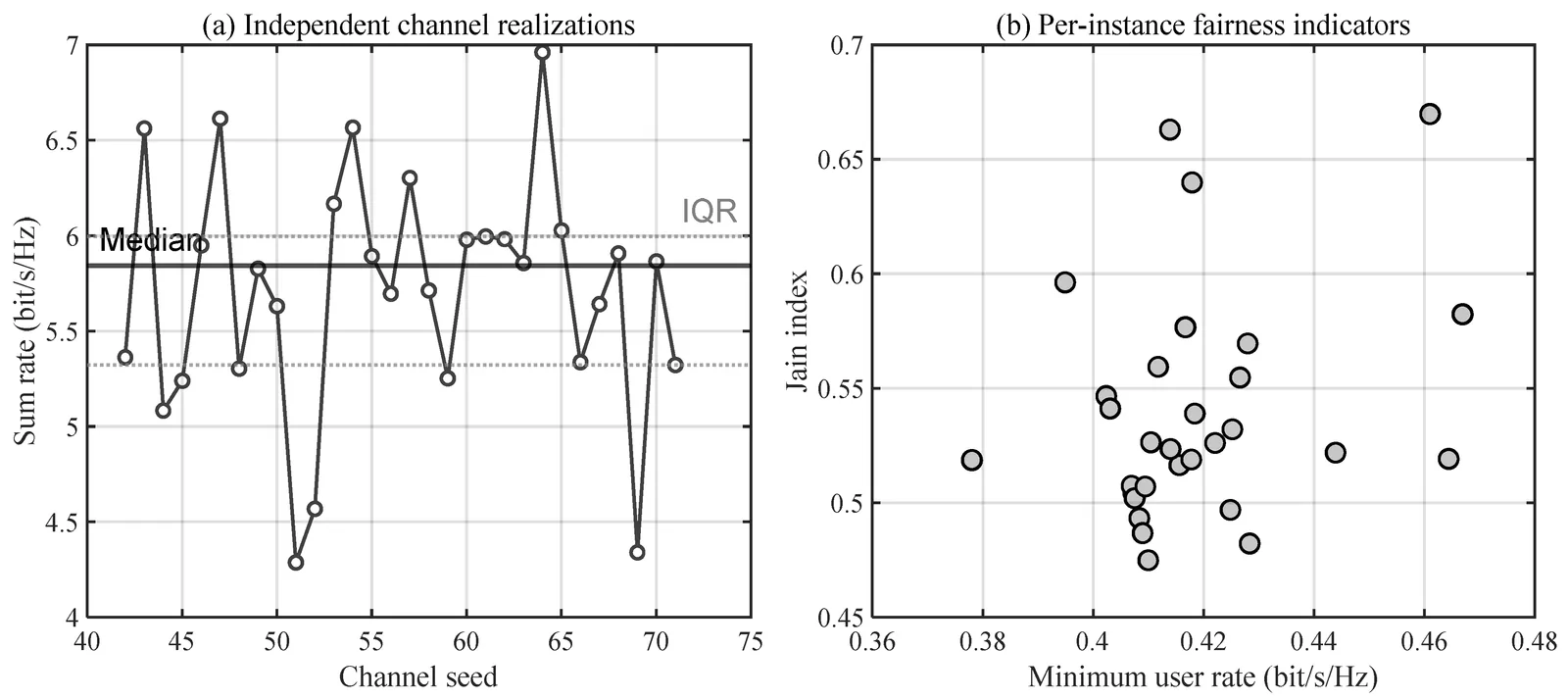
Thirty-channel JODS-PD results. (**a**) Returned sum rates with the median and interquartile-range boundaries; (**b**) per-instance Jain index versus minimum-user rate.

The nonparametric bootstrap 95% confidence interval for the median sum rate is [5.4958, 5.9637] bit/s/Hz. Across the 30 endpoints, the maximum scaled constraint violation is 4.32 × 10^−10^ and the maximum flow-residual infinity norm is 8.73 × 10^−8^. Figure 6 visualizes the returned sum rates over the 30 independent channel realizations and the relationship between the minimum-user rate and the Jain index.

### 4.5. Connection to the Theory and Scope of the Evidence

For each terminal state, four standard KKT residuals were evaluated:

(30) $$\begin{array}{cc} r_{stat} & =∥\nabla f+J_{h}^{T}\mu {∥}_{\infty },\\ r_{pri} & =∥[\text{h}{]}_{+}{∥}_{\infty },\\ r_{dual} & =∥[-\mu {]}_{+}{∥}_{\infty },\\ r_{comp} & =∥\mu ⊙\text{h}{∥}_{\infty }. \end{array}$$

For the four residuals in (30), the largest values over seeds 42–51 were *r*_stat_ = 8.791 × 10^−8^, *r*_pri_ = 4.307 × 10^−10^, *r*_dual_ = 1.680 × 10^−14^, and *r*_comp_ = 4.206 × 10^−10^, respectively. In particular, the maximum primal feasibility residual was well below the 10^−6^ feasibility criterion. These quantities, together with the flow residual, support the claim that the returned states are approximate KKT equilibria under Proposition 2. They do not by themselves establish the local assumptions of Theorem 1.

We also evaluated the symmetry-aware quantities in Table 7. The active branch is identified from the projected multiplier map. The tangent dimension combines one phase direction per nonzero stream and the active-multiplier fiber dimension; the remaining eigenvalues are classified as transverse. This decomposition directly tests the geometric objects appearing in Theorem 1.

The active Jacobians are rank deficient by one or two, so LICQ does not hold at these endpoints. The missing ranks generate multiplier fiber directions, but they do not invalidate transverse set attraction when the rank is locally constant and the quotient normal block remains positive. The observed zero-mode counts agree with the predicted phase-plus-fiber tangent dimensions in all ten matched cases.

These are finite-precision pointwise checks rather than numerical proof of the neighborhood assumptions. Seed 49 has the smallest positive quotient curvature and the least negative transverse spectral margin, making it the most weakly conditioned matched endpoint. The result is consistent with, but does not replace, the local hypotheses of Theorem 1.

### 4.6. Decoding Order and Multi-Start Sensitivity

**Figure 7:**
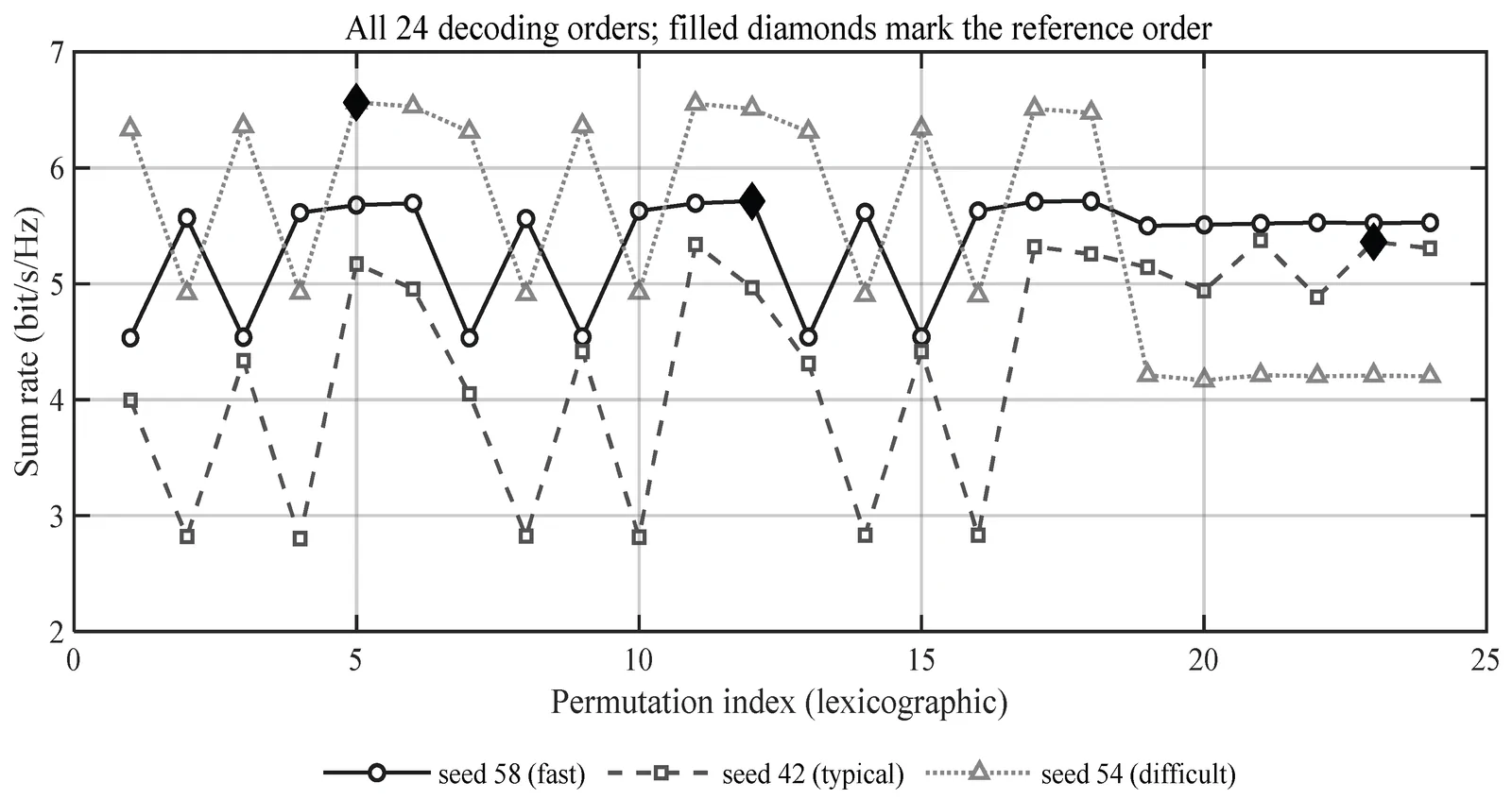
Decoding order sensitivity for seeds 58, 42, and 54. The three curves show the returned sum rates across all 24 permutations and identify the prescribed reference order.

All 24 decoding orders were enumerated for seeds 58, 42, and 54. The reference order ranked first, second, and first, respectively, among successful orders. The successful sum-rate ranges were 4.5329–5.7122, 2.8028–5.3747, and 4.2027–6.5651 bit/s/Hz. Across the 30-channel study, the final effective-gain order matched the initial reference order in 28 cases, indicating that the fixed policy is usually self-consistent but not universally best. The corresponding sum-rate variation across all 24 decoding orders is illustrated in Figure 7, where the prescribed reference order is highlighted for each representative channel. The exhaustive decoding-order results for the three representative channels are summarized in Table 8.

To examine local solution sensitivity separately from decoding order, six initializations were tested for each of seeds 42, 47, and 54 while keeping the order fixed. All 18 runs met the endpoint criterion. Seeds 47 and 54 converged to numerically identical objective values, whereas seed 42 produced two nearby values, 5.3248 and 5.3620 bit/s/Hz. Thus, the tested starts show high repeatability but do not establish global optimality. The fixed-order multi-start results, including the successful-start counts and returned sum-rate ranges, are summarized in Table 9.

### 4.7. Parameter Sensitivity and Conditioning

The principal numerical parameters were varied on three representative channels. Moderate changes in ρ, γ, warm-up length, Jacobian refresh period, and stopping tolerance generally preserve the returned endpoint, but they can alter iteration counts markedly. In particular, refreshing the generalized Jacobian every iteration reduces the difficult seed 54 count from 2446 to 227, showing that its long trajectory is mainly a derivative reuse and active-branch conditioning effect rather than a failure of the equilibrium formulation. The tested parameter values, successful-run counts, and principal numerical observations are summarized in Table 10.

**Figure 8:**
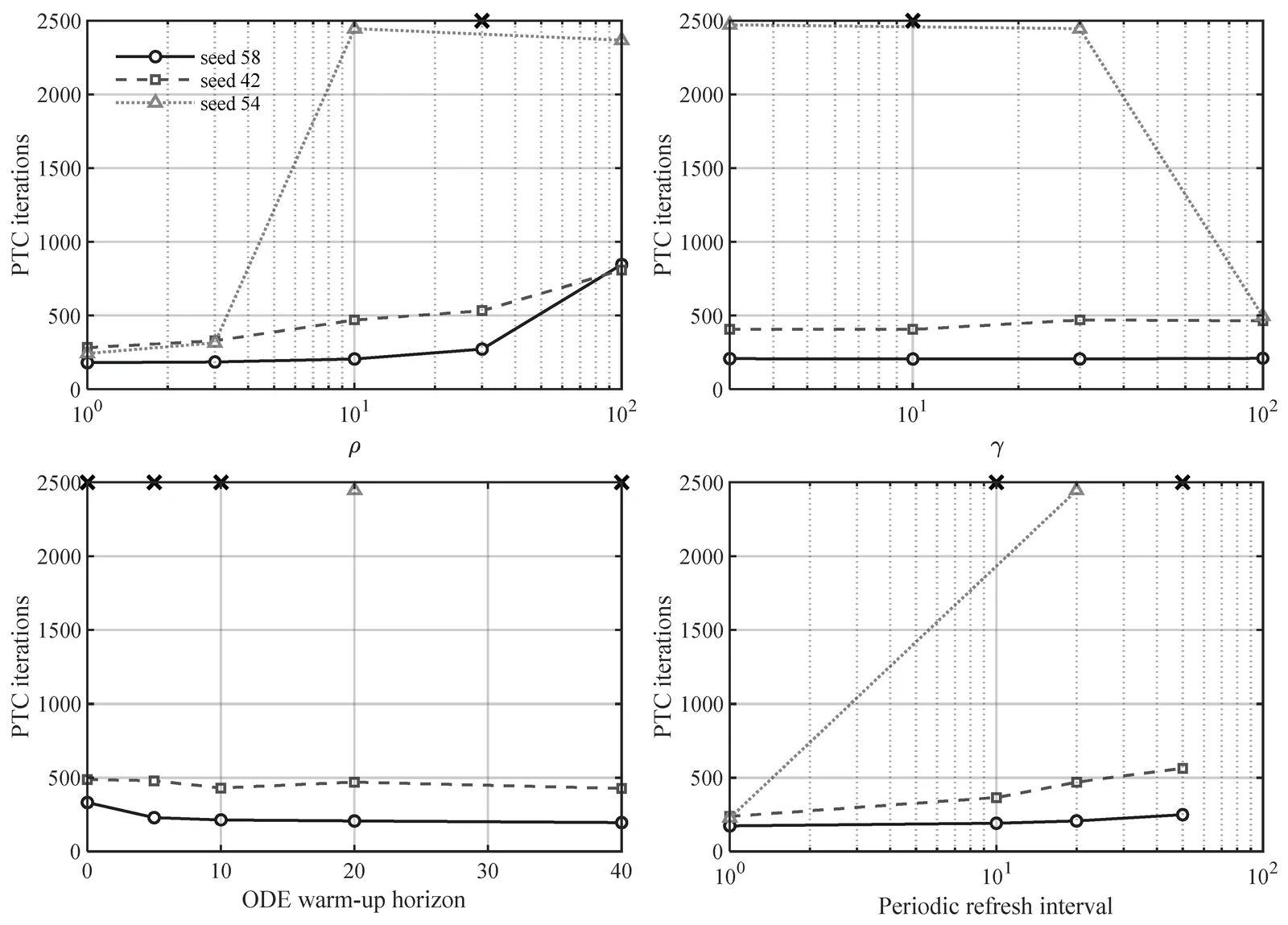
Parameter sensitivity of the PTC iterations. Solid, dashed, and dotted curves represent seeds 58, 42, and 54, respectively. Crosses indicate unsuccessful runs, while light-gray solid and dotted vertical lines denote major and minor gridlines, respectively.

For γ = 3, 10, 30, and 100, the difficult seed required 2472, more than 2500, 2446, and 494 PTC iterations, respectively. This supports the theorem’s qualitative statement that any γ > 0 preserves local transverse stability under its assumptions, while the numerical conditioning and convergence speed can still depend strongly on γ. The main effects of the numerical parameters on the PTC iteration count and convergence behavior are illustrated in Figure 8.

### 4.8. Finite-Size Scalability and Structured Implementation

One-factor and combined-size tests were used to assess finite instance computational behavior. The structured implementation assembled the exact primal–dual block Jacobian and eliminated strictly inactive multipliers from the shifted solve. On 27 pre-specified benchmark instances, it succeeded in all 27 cases, compared with 25 for the dense realization, while the maximum absolute sum-rate difference on common successes was 6.18 × 10^−11^ bit/s/Hz. At the largest one-factor settings, the median speedups were 3.36× for M = 100, 2.98× for K = 6, and 7.69× for N = 8. The success rates and median solver times of the structured implementation on the selected finite-size instances are reported in Table 11.

**Figure 9:**
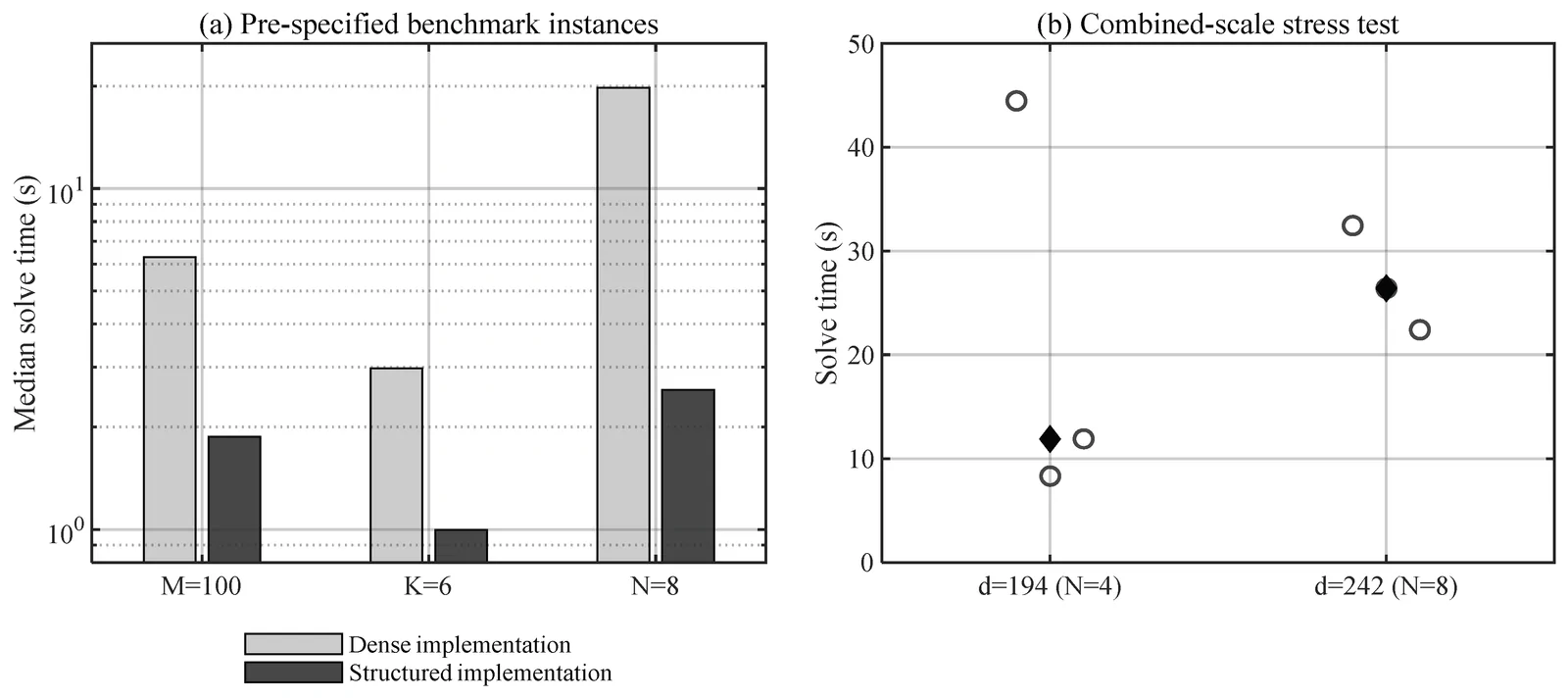
Effects of structured Jacobian assembly and inactive multiplier elimination. (**a**) Median solver time for the pre-specified M = 100, K = 6, and N = 8 one-factor instances; (**b**) per-run solver time for the combined d = 194 and d = 242 stress tests. In panel (**b**), open circles represent individual runs, and filled diamonds indicate the median solve times.

The two combined settings (N, M, K) = (4, 100, 6) and (8, 100, 6) both succeeded in all three saved channel realizations. Across them, the largest final flow residual was 9.78 × 10^−8^ and the largest scaled constraint violation was 2.66 × 10^−10^. These results demonstrate practical solvability for the tested finite instances up to d = 242 and identify structured linear algebra as the main route to further reductions in computation time. The main effects of the numerical parameters on the PTC iteration count and convergence behavior are illustrated in Figure 9.

### 4.9. Imperfect CSI Stress Test

The estimated effective channels were perturbed at NMSE levels of −30, −20, and −10 dB. JODS-PD converged on all 30 estimated-channel problems, but none of the corresponding true-channel evaluations remained feasible at the stringent 10^−6^ threshold. The median raw sum-rate changes were −0.061%, 0.169%, and 3.858%, while the median maximum true-channel constraint violations increased from 2.93 × 10^−2^ to 1.73 × 10^−1^. Thus, objective values can appear stable even when SIC or received-power constraints are no longer robust. The convergence results, raw sum-rate changes, true-channel constraint violations, and decoding-order changes under imperfect CSI are summarized in Table 12.

**Figure 10:**
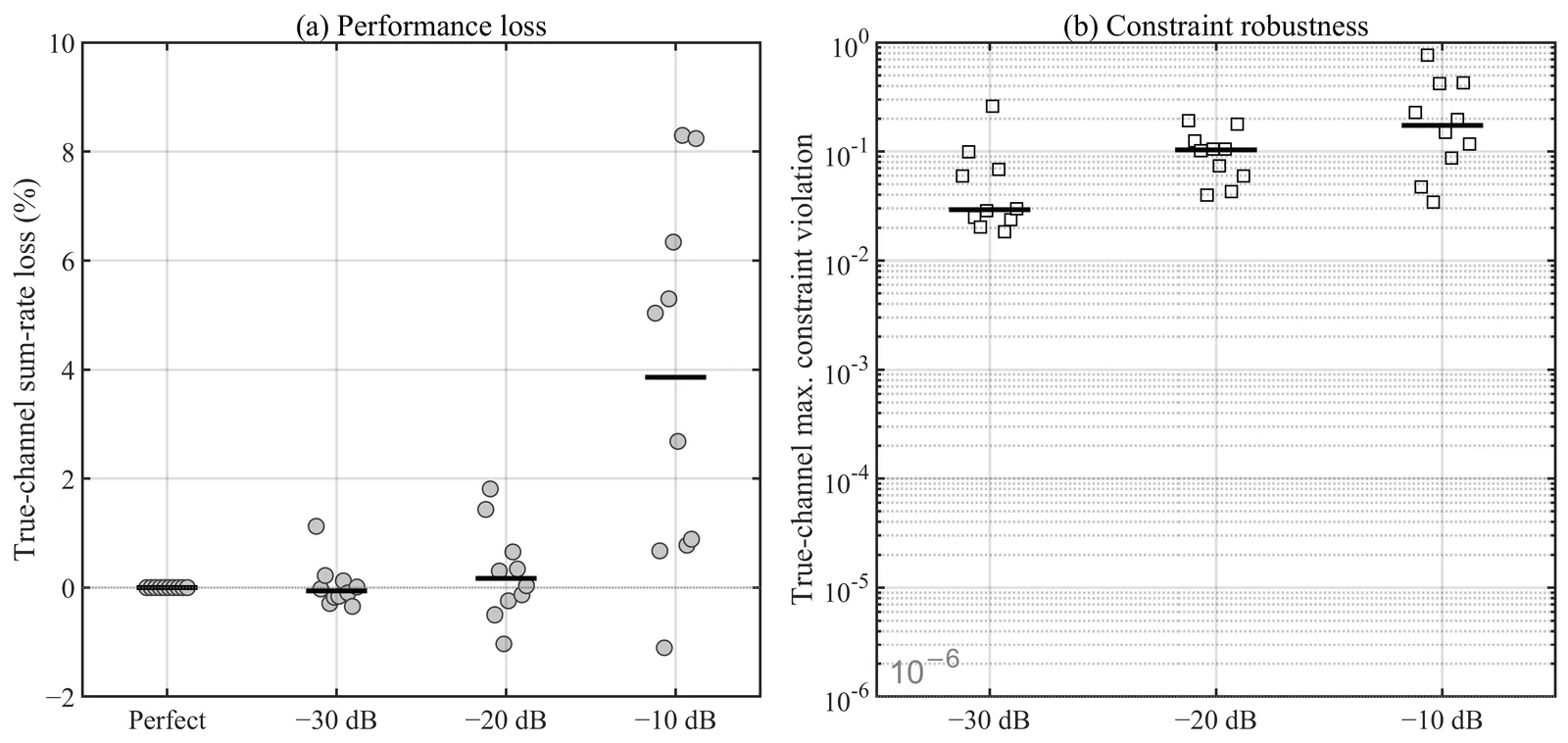
Imperfect CSI stress test. (**a**) True-channel sum-rate change relative to the perfect-CSI reference; (**b**) maximum true-channel constraint violation at −30, −20, and −10 dB NMSE. Horizontal bars indicate medians. Circles in panel (**a**) represent individual true-channel sum-rate losses, while squares in panel (**b**) represent individual maximum constraint violations.

The order-change counts were 2/10, 1/10, and 8/10 from −30 to −10 dB. This sensitivity indicates that robust or chance-constrained CSI treatment should protect both the continuous beamforming variables and the discrete order decision. The present stress test is diagnostic; it does not replace a dedicated robust optimization model. The resulting true-channel sum-rate changes and maximum constraint violations at the three NMSE levels are shown in Figure 10.

## 5. Discussion, Practical Implications, and Limitations

The results support three distinctions. First, single-layer describes simultaneous primal–dual optimization, whereas ODE warm-up and PTC are numerical stages for the same root map. Second, local transverse attraction concerns distance to a non-isolated KKT set rather than pointwise asymptotic stability. Third, endpoint feasibility and stationarity should be reported separately: AO-IPOPT is a strong same-model feasible baseline, while JODS-PD is designed to drive the full primal–dual residual to a low level.

In a deployment, the controller would alternate between channel acquisition and optimization across coherence blocks, while distributed implementations may additionally require reliable synchronization among networked nodes [47]. Explicit channel estimation provides physical CSI and interpretable error statistics [24], whereas implicit or learning-assisted acquisition can reduce online reconstruction cost at the price of training data and distribution shift dependence [25] and may additionally raise privacy concerns when user or network data are involved [48]. Table 13 summarizes how these choices interface with the present optimization layer.

The finite-size results suggest several extensions. Minimum-rate, weighted-utility, and fairness constraints can be added within the same projected primal–dual framework. Dynamic or globally optimized decoding order requires an outer combinatorial, relaxation, or learning-based mechanism because the current continuous theorem assumes a locally fixed order. Multi-cell interference, discrete phase shifts, STAR-RIS modes, hardware loss, and mobility introduce additional constraints or nonsmooth mode choices [20,21,49]. Energy prediction coupled with optimized routing has also been investigated [50], suggesting a possible extension toward energy-aware control across mobile or intermittently connected nodes. Robust CSI treatment should be integrated into the formulation rather than inferred from nominal objective stability. Distributed deployment also requires security at both controller and data-plane levels. Recent studies have proposed machine learning mitigation for low-rate flow-table overflow [51], multi-window detection and collaborative flow-rule mitigation [52], programmable-data-plane defenses against bounce-style DNS DDoS attacks [53], and deep-learning-based attack detection with authentication mechanisms [54]. These measures complement broader zero-trust protection architectures [55].

The evidence remains bounded by the tested channel model, finite dimensions, software implementation, and local non-convex endpoints. The order and multi-start studies do not prove global optimality; the pointwise spectral diagnostics do not verify all neighborhood hypotheses of Theorem 1; and the scalability results do not establish dimension-independent or asymptotic complexity. These limitations define the scope of the reported conclusions and motivate sparse/iterative linear solvers, adaptive derivative refresh, robust constraints, and outer order optimization.

## 6. Conclusions

JODS-PD formulates fixed-order IRS-assisted NOMA beamforming as one coupled projected primal–dual state after exact angle parameterization and equivalent adjacent-order reduction. The analysis establishes equilibrium–KKT equivalence and local transverse exponential attraction of the non-isolated KKT set under explicit regularity, quotient curvature, and finite penalty assumptions. The hybrid stiff-ODE/semismooth-PTC realization consistently produced low-residual endpoints on the 30 tested baseline channels. Same-model AO-IPOPT and MIPS comparisons, together with order, multi-start, sensitivity, fairness, finite-size scalability, and imperfect CSI tests, clarify where the method performs reliably and where conditioning, discrete order selection, or model uncertainty remains important. The largest tested state dimension was 242, for which structured Jacobian assembly and inactive multiplier elimination achieved 3/3 successes with a median solver time of 26.40 s. Future work will focus on robust CSI-aware constraints, adaptive decoding order mechanisms, sparse or iterative PTC linear algebra, and extensions to larger multi-cell and hardware-constrained IRS architectures.

## Figures and Tables

**Table 1:** Positioning of the compared numerical approaches.

Approach	Variable Update	IRS and Constraints	Endpoint/Theory Statement	Main Implementation Burden
Representative IRS–NOMA AO/SCA/SDR	Alternating or block sequential	Relaxation/approximation with block constraints	Typically block-stationary or locally stationary under method-specific assumptions	Repeated convex/subproblem solves and recovery
MATPOWER Interior Point Solver (MIPS)	All variables in a generic interior-point NLP	Explicit nonlinear constraints	Solver termination and returned primal feasibility	Generic KKT factorization; scaling depends on formulation
AO-IPOPT	Two exact nonlinear blocks	Angle-parameterized IRS; exact block constraints	Outer convergence and primal feasibility; block-stable endpoint	Repeated exact-derivative IPOPT solves
JODS-PD	Simultaneous primal–dual state	Angle coordinates and adjacent ordering	Equilibrium–KKT equivalence and local transverse attraction of the KKT set	Active-set semismooth PTC and endpoint KKT audit

**Table 2:** System and channel parameters used in the matched benchmark.

Parameter	Value
BS antennas, N	2
IRS elements, M	30
Users, K	4
BS location	(5,0,0) m
IRS location	(0,50,0) m
User region	radius-3 m disk centered at (50,5,0) m
Transmit power, $P_{T}$	10 dBm (0.01 W)
Reference path gain, $\beta_{0}$	−30 dB at 1 m
Path-loss exponents, $\alpha_{D}/\alpha_{BI}/\alpha_{IU}$	3.5/2.2/2.2
Rician factors, $\kappa_{BI},\kappa_{IU}$	3 dB
Bandwidth, B	1 MHz
Noise power spectral density	−150 dBm/Hz
Linear noise power spectral density, $N_{0}$	$10^{(-150-30)/10}=10^{-18}$ W/Hz
Noise power, $\sigma^{2}=BN_{0}$	$10^{-12}$ W
Independent channel realizations	30 independent channel realizations; seeds 42–51 used for matched solver comparisons

**Table 3:** Principal numerical settings for JODS-PD and MIPS.

Setting	JODS-PD	MIPS
Penalty/dual gain	ρ=10, γ=30	—
Stiff warm-up	ode15s on t∈[0,20]; 300 event-call cap, optionally extended by 500	—
Warm-up tolerances	RelTol $10^{-3}$, AbsTol $10^{-6}$	—
Warm-up maximum step	MATLAB default	—
Pilot-residual extension threshold	100	—
PTC scaling	c=0.6, $\varepsilon_{b}=10^{-2}$	—
Generalized-derivative refresh	20 steps, plus active-set and near-solution refreshes	Exact callbacks every iteration
Equilibrium stopping rule	${∥F∥}_{2}\leq 10^{-7}$ or ${∥F∥}_{\infty }\leq 10^{-7}$	—
Iteration/time limit	2500 PTC steps	500 interior-point iterations
Nonlinear feasibility/optimality tolerances	—	$10^{-6}$
Step control	Nonmonotone SER update	Enabled
Hessian regularization	None	$10^{-8}I$

**Table 4:** Representative convergence and generalized Jacobian refresh statistics.

Case	Seed	PTC Iterations	Scheduled	Active-Set	Near-Root	Final ||F||∞
Fast	58	206	11	0	5	1.54 × 10^−8^
Typical	42	470	24	47	8	5.30 × 10^−9^
Slow	48	774	39	102	13	2.17 × 10^−8^
Difficult	54	2446	123	2237	5	2.79 × 10^−11^

**Table 5:** Matched comparison over seeds 42–51.

Metric	JODS-PD	AO-IPOPT	MIPS
Successful endpoints/10	10	10	8
Median sum rate on 8 common successes (bit/s/Hz)	5.7285	5.6923	5.2145
Median KKT/stationarity residual on common successes	<10^−6^	approximately 7 × 10^−3^ (reconstructed full KKT)	method-dependent
Update architecture	simultaneous primal–dual	two-block alternating	generic interior point
Returned endpoint interpretation	low-residual KKT point	primal feasible block-stable point	primal feasible solver endpoint

**Table 6:** Distributional and user-level statistics for 30 JODS-PD channel realizations.

Metric	Mean ± Standard Deviation	Median [IQR]	Range
Sum rate (bit/s/Hz)	5.7073 ± 0.6307	5.8420 [5.3259, 5.9919]	[4.2867, 6.9603]
Minimum-user rate (bit/s/Hz)	0.4188 ± 0.0193	0.4148 [0.4085, 0.4251]	[0.3780, 0.4669]
Jain index	0.5399 ± 0.0498	0.5248 [0.5072, 0.5582]	[0.4749, 0.6698]
PTC iterations	582.0 ± 391.8	500 [398.75, 661]	[207, 2447]
Solver time (s)	0.5531 ± 0.9259	0.3901 [0.2816, 0.4518]	[0.1670, 5.3594]

**Table 7:** Symmetry-aware endpoint diagnostics over seeds 42–51.

Diagnostic	Observed Range/Count	Connection to Theorem 1
Minimum quotient Hessian eigenvalue	9.02 × 10^−5^ to 1.88 × 10^−3^	positive curvature on tested quotient-critical subspaces
Transverse spectral abscissa	−1.88 × 10^−3^ to −8.91 × 10^−5^	negative real parts in the complementary subspace
Phase-tangent residual	at most 1.56 × 10^−9^	numerical invariance of the phase orbit
Active-rank deficiency	1 or 2	nontrivial multiplier fiber tangent directions
Observed zero modes	equal to predicted tangent dimension in all 10 cases	phase orbit plus multiplier fiber neutrality
Borderline curvature case	seed 49	small but positive transverse margin

**Table 8:** Exhaustive order enumeration on three representative channels.

Seed	Reference Order Rank	Successful Order Sum-Rate Range (bit/s/Hz)	Interpretation
58	1/24	4.5329–5.7122	reference policy attains the best successful order
42	2/24	2.8028–5.3747	one alternative order is better
54	1/24	4.2027–6.5651	reference policy attains the best successful order

**Table 9:** Fixed-order multi-start results.

Seed	Successful Starts	Returned Sum-Rate Range (bit/s/Hz)	Within-Cluster Numerical Gap
42	6/6	5.3248421–5.3620263	7.4 × 10^−10^%
47	6/6	6.6135325–6.6135325	7.5 × 10^−12^%
54	6/6	6.5651223–6.5651223	3.5 × 10^−11^%

**Table 10:** Summary of the parameter sensitivity study (three channels per setting).

Parameter	Tested Values	Observed Successful Runs	Main Numerical Observation
Penalty ρ	1, 3, 10, 30, 100	2/3 to 3/3	ρ = 100 gives median 847 iterations
Dual gain γ	3, 10, 30, 100	2/3 to 3/3	changes time-scale balance and conditioning
Warm-up length	0, 5, 10, 20, 40	2/3 to 3/3	a short warm-up reduces poor initial branch models
Jacobian period	1, 10, 20, 50	2/3 to 3/3	seed 54: 2446 to 227 iterations at period 1
Flow/KKT tolerance	10^−6^, 10^−7^, 10^−8^	3/3 for each	endpoint residuals track the requested tolerance

**Table 11:** Structured JODS-PD on selected finite-size instances.

N	M	K	State Dimension d	Successes	Median Solver Time (s)
2	100	4	135	3/3	1.87
2	30	6	100	3/3	1.00
8	30	4	113	3/3	2.57
4	100	6	194	3/3	11.91
8	100	6	242	3/3	26.40

**Table 12:** Imperfect CSI evaluation on ten channels per NMSE level.

NMSE (dB)	Estimated Problem Convergence	Median Raw Sum-Rate Change	Median True Max. Violation	Order Changes
−30	10/10	−0.061%	2.93 × 10^−2^	2/10
−20	10/10	0.169%	1.03 × 10^−1^	1/10
−10	10/10	3.858%	1.73 × 10^−1^	8/10

**Table 13:** CSI acquisition choices and their interaction with JODS-PD.

CSI Route	Information Supplied To Optimizer	Principal Advantage	Principal Design Issue
Explicit pilot-based estimation	direct and cascaded channel estimates with error statistics	physical interpretability and compatibility with robust constraints	training overhead increases with IRS/user dimensions
Implicit/learning-assisted acquisition	features, effective channels, or direct configuration proposals	potentially lower online estimation latency	training coverage, generalization, and uncertainty calibration
Present paper	one quasi-static channel estimate per optimization block	isolates primal–dual numerical behavior	acquisition overhead excluded; robustness assessed only by stress test

## Data Availability

The raw data supporting the conclusions of this article will be made available by the authors on request.
